# OnabotulinumtoxinA (Botox) in the Treatment of Crow’s Feet Lines in Japanese Subjects

**DOI:** 10.1007/s00266-017-0844-9

**Published:** 2017-07-21

**Authors:** Kiyonori Harii, Makoto Kawashima, Nobutaka Furuyama, Xiaofang Lei, René Hopfinger, Elisabeth Lee

**Affiliations:** 1 Department of Plastic Surgery, Kyorin University School of Medicine, 6-20-2 Shinkawa, Mitaka, Tokyo 181-0004 Japan; 2 Department of Dermatology, Tokyo Women’s Medical University School of Medicine, Tokyo, Japan; 3 Jiyugaoka Clinic, Tokyo, Japan; 4 Allergan plc, Irvine, CA USA

**Keywords:** Skin aging, Botox, Botulinum toxins, type A, Neurotoxins

## Abstract

**Background:**

This study evaluated the safety and efficacy of onabotulinumtoxinA in Japanese subjects with crow’s feet lines (CFL).

**Methods:**

This phase 3, multicenter, double-blind, randomized study included 2 treatment periods: 6-month placebo-controlled period followed by a 7-month open-label period. In period 1, subjects with moderate to severe CFL received onabotulinumtoxinA 24 U (*n* *=* 104) or 12 U (*n* *=* 99), or placebo (*n* *=* 97). In period 2, placebo subjects switched to onabotulinumtoxinA 24 U or 12 U (double-blind dose). Up to 5 total treatments were permitted for subjects meeting re-treatment criteria. The primary efficacy measure was the proportion of investigator-assessed responders (achieving CFL severity of none or mild at maximum smile using the Facial Wrinkle Scale with Asian Photonumeric Guide [FWS-A] at day 30 of treatment 1). Additional endpoints included other responders (achieving at least 1-grade improvement at maximum smile and at rest using the FWS-A at day 30), responders at other time points, duration of effect, subject-reported outcomes, and safety.

**Results:**

All efficacy endpoints were met. At day 30, the proportion of subjects achieving none or mild severity at maximum smile was significantly greater (*P* < 0.001) in the onabotulinumtoxinA 24 and 12 U groups (68.3 and 56.6%, respectively) compared with the placebo group (8.2%). Efficacy results were consistent over repeated treatments, and subjects’ self-assessed outcomes were similar to investigator-assessed results.

**Conclusions:**

Treatment with onabotulinumtoxinA 24 and 12 U improved the appearance of CFL in Japanese subjects and was well tolerated, with no new safety findings.

**Level of Evidence I:**

This journal requires that authors assign a level of evidence to each article. For a full description of these Evidence-Based Medicine ratings, please refer to the Table of Contents or the online Instructions to Authors www.springer.com/00266.

## Introduction

The use of onabotulinum toxin type A injections for minimally invasive esthetic procedures has become globally popular [1]. OnabotulinumtoxinA (Botox Cosmetic; Allergan plc, Dublin, Ireland) is approved for the treatment of glabellar lines (GL) in more than 70 countries, including Japan, and for the treatment of crow’s feet lines (CFL) in more than 50 countries, including the USA and countries in the European Union.

The efficacy of onabotulinumtoxinA for treatment of CFL has been demonstrated in trials conducted in predominantly non-Asian populations [2, 3]. In these trials, improvement in CFL severity with onabotulinumtoxinA treatment was evaluated using the Facial Wrinkle Scale (FWS; Allergan plc, Dublin, Ireland) [2, 3]. However, it has been noted that Asian facial skin has increased dermal thickness, collagen content, and melanin [4–6], and assessment of CFL morphology in Asian subjects has shown slightly different CFL pattern prevalence compared with predominantly Caucasian subjects [5, 6]. For these reasons, the FWS with Asian Photonumeric Guide (FWS-A) was designed to provide clinicians with a standardized, static method for assessing the severity of facial lines in Asian subjects. The present study evaluated the safety and efficacy of onabotulinumtoxinA compared with placebo in Japanese subjects with moderate to severe CFL, with severity investigator-assessed using the validated FWS-A.

## Materials and Methods

### Study Design

This 13-month, phase 3, multicenter, double-blind, randomized, parallel-group study comprised 2 treatment periods: a 6-month placebo-controlled treatment period followed by a 7-month open-label period. Two doses of onabotulinumtoxinA (24 and 12 U) were evaluated for the treatment of moderate to severe CFL. These doses were compared with placebo in treatment period 1 (up to day 180), which consisted of an initial treatment (day 1) followed by up to 1 additional treatment. In treatment period 2 (starting on study day 180), all subjects received either onabotulinumtoxinA 24 U or onabotulinumtoxinA 12 U (up to 3 re-treatments) to assess the safety and efficacy of repeated treatments through the end of the study (day 390). All subjects could receive up to 5 total treatments based on meeting re-treatment criteria. The onabotulinumtoxinA 24 and 12 U doses were administered in a double-blind manner throughout the study.

### Study Treatments

**Fig. 1:**
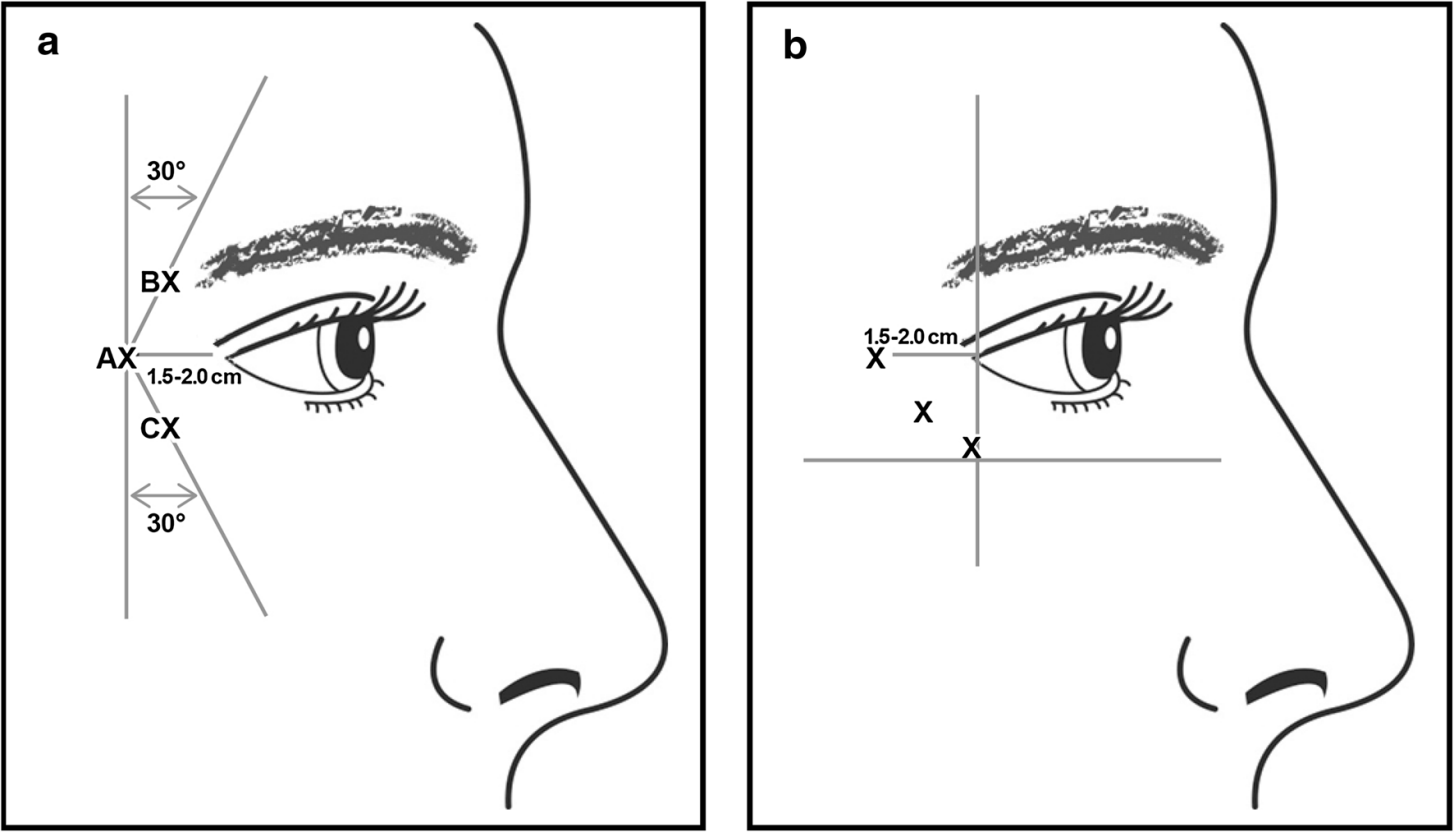
Alternative injection patterns for treatment of crow’s feet lines. **a** The first injection was in the orbicularis oculi at the level of the lateral canthus, at least 1.5–2.0 cm temporal to the lateral canthus and just temporal to the lateral orbital rim (marked as AX). The second injection was 1.0–1.5 cm above this first injection site, at an approximately 30° angle medially (marked as BX). The third injection was 1.0–1.5 cm below the first injection site, at an approximately 30° angle medially (marked as CX). **b** If the lines in the crow’s feet region were primarily below the lateral canthus, the injector had the option to inject below the lateral canthus. Injections were given in a line angling from anteroinferior to superoposterior, with the most anterior injection point lateral to a line drawn vertically from the lateral canthus and the most inferior injection superior to the maxillary prominence. Reprinted with permission from Carruthers et al. [2]

Eligible subjects were randomized at the day 1 visit to 1 of the following 4 treatment groups in a 2:2:1:1 ratio for treatment periods 1 and 2: onabotulinumtoxinA 24 U/onabotulinumtoxinA 24 U, onabotulinumtoxinA 12 U/onabotulinumtoxinA 12 U, placebo/onabotulinumtoxinA 24 U, or placebo/onabotulinumtoxinA 12 U. Subjects were assigned a randomization number (not disclosed to the study center), and randomization was stratified by baseline CFL severity at maximum smile. The study drug was packaged and labeled with kit numbers; each kit contained 1 vial of study drug. An interactive voice or web response system designed by Allergan Data Management provided a specific kit number for each subject, and the study center administered treatment. The same procedure was followed during subsequent study visits. During treatment period 2, subjects who received onabotulinumtoxinA in period 1 continued to receive the same treatment dose, and subjects who received placebo in period 1 received either onabotulinumtoxinA 24 U or onabotulinumtoxinA 12 U, as determined on randomization at day 1. Each treatment comprised a total of 6 intramuscular injections in the lateral aspect of the orbicularis oculi (3 injections per side; 0.1 mL per injection site). Two alternative treatment patterns (Fig. 1) were available to the investigators, who selected a pattern based on the subject’s pattern of CFL and on their own clinical judgment.

Each subject could receive up to 5 treatments over the 13-month study period, based on re-treatment criteria (1 initial treatment and up to 1 additional treatment in period 1; up to 3 additional treatments in period 2). Re-treatment was permitted if subjects met all of the following criteria: bilateral CFL of at least moderate severity at maximum smile, as measured by the investigator using the FWS-A; a negative pregnancy test; and at least 3 months since the last treatment (no earlier than 84 days); no treatment was allowed after the day 360 visit.

### Subjects

Eligible subjects were Japanese males and nonpregnant females aged 20–64 years, with bilaterally symmetrical moderate to severe CFL at maximum smile, as measured by the investigator using the FWS-A. Subjects with previous cosmetic treatments or surgical procedures at the treatment sites were excluded. Other key exclusion criteria included eyebrow or eyelid ptosis and eyelid hooding or other skin laxity likely to interfere with onabotulinumtoxinA treatment or CFL assessments.

This study was conducted in compliance with the International Council for Harmonisation guidelines and Japanese Good Clinical Practice guidelines, as well as applicable US Food and Drug Administration guidelines. All subjects provided written informed consent prior to study participation.

### Assessments and Measures

Subjects attended up to 24 visits (depending on the number of treatments received). Visits included randomization/treatment (day 1); follow-up (at weeks 1 and 2 following each treatment); monthly from months 1 through 12; and upon exit (month 13 or early discontinuation). Differences in duration of response between subjects resulted in differences in the timing to re-treatment eligibility; accordingly, the study design allowed treatment/re-treatment intervals of various potential duration (Table 1). On treatment days, all measurements were taken prior to treatment.

**Table 1:** Potential maximum duration of treatment intervals

Treatment	Duration (days)
1	≤390 (day 1 treatment in period 1)
2	≤300 (second treatment in period 1 or first treatment in period 2)
3	≤210 (first or second treatment in period 2)
4	≤120 (second or third treatment in period 2)
5	≤30 (third treatment in period 2)

The primary efficacy measure was the proportion of investigator-assessed responders at day 30 after initial treatment, with responders defined as subjects achieving CFL severity of none or mild severity at maximum smile on the FWS-A (0 *=* none, 1 *=* mild, 2 *=* moderate, and 3 *=* severe). Additional efficacy endpoints based on the investigator-assessed FWS-A included the following: the proportion of subjects achieving at least a 1-grade improvement in CFL severity at maximum smile and at rest (responders) at day 30; the duration of effect, defined as the median time until loss of efficacy (from responder to nonresponder in treatment period 1 for day 30 responders), using the following FWS-A responder definitions: CFL severity of none or mild at maximum smile, at least a 1-grade improvement in CFL severity at maximum smile, and ≥1-grade improvement in CFL severity at rest; and responder analyses at time points other than day 30 of treatment period 1.

Several subject-reported outcomes were also assessed. The subject’s global assessment of change in CFL (SGA-CFL) was evaluated on a 7-point scale ranging from 1 (very much improved) to 7 (very much worse). On the self-perception of age (SPA), subjects reported if they perceived themselves as looking their current age, older than their current age, or younger than their current age. Subjects’ perception of the effect of their facial lines on their appearance was assessed using specific items from the 11-item Facial Line Outcomes (FLO-11) Questionnaire including the psychological impact items 2 (“look older”), 5 (“look less attractive”), and 8 (“look tired”). Responses were based on a scale wherein 0 indicates “not at all” and 10 indicates “very much”; responders were subjects achieving at least a 2-point improvement for items 2 and 5 and at least a 3-point improvement for item 8. Subject Assessment of Satisfaction with Appearance was based on a 5-point scale (1 *=* very unsatisfied; 5 *=* very satisfied), and a responders were defined as subjects who rated their satisfaction as improved (ie, from neutral or worse at baseline to very satisfied or satisfied after treatment). Satisfaction with treatment was assessed by the Facial Line Satisfaction Questionnaire (FLSQ) overall satisfaction item. Responses were based on a 5-point Likert scale ranging from −2 (very dissatisfied) to +2 (very satisfied); responders were subjects who were “mostly” or “very” satisfied. Finally, subjects’ perception of onset of effect was assessed by asking subjects at weeks 1 and 2 if they noticed an improvement in CFL appearance; those who answered yes were asked when (in number of days) the improvement was first noticed.

Safety measures were adverse events (AEs), vital sign measurements (pulse rate, respiratory rate, and blood pressure), physical examination, urine pregnancy test for female subjects of childbearing potential, and neurologic assessments (focused symptoms questionnaire and focused neurologic examination).

### Statistical Analyses

Analyses were performed on the intent-to-treat population (all subjects who were randomized) and safety population (all subjects who received ≥1 injection of study drug). The last observation carried forward method was used for imputation of missing values through day 90 of each treatment. Between-group baseline and demographic comparisons were performed using the Kruskal–Wallis test. For the FWS-A, FLO-11, satisfaction with treatment (FLSQ), and satisfaction with appearance, the comparison of the proportion of responders was performed using the Cochran–Mantel–Haenszel test stratified by baseline CFL severity at maximum smile. The median time to onset of improvement in CFL as reported by subjects at weeks 1 and 2 during each treatment period was estimated using the Kaplan–Meier survival method.

## Results

### Subjects

**Fig. 2:**
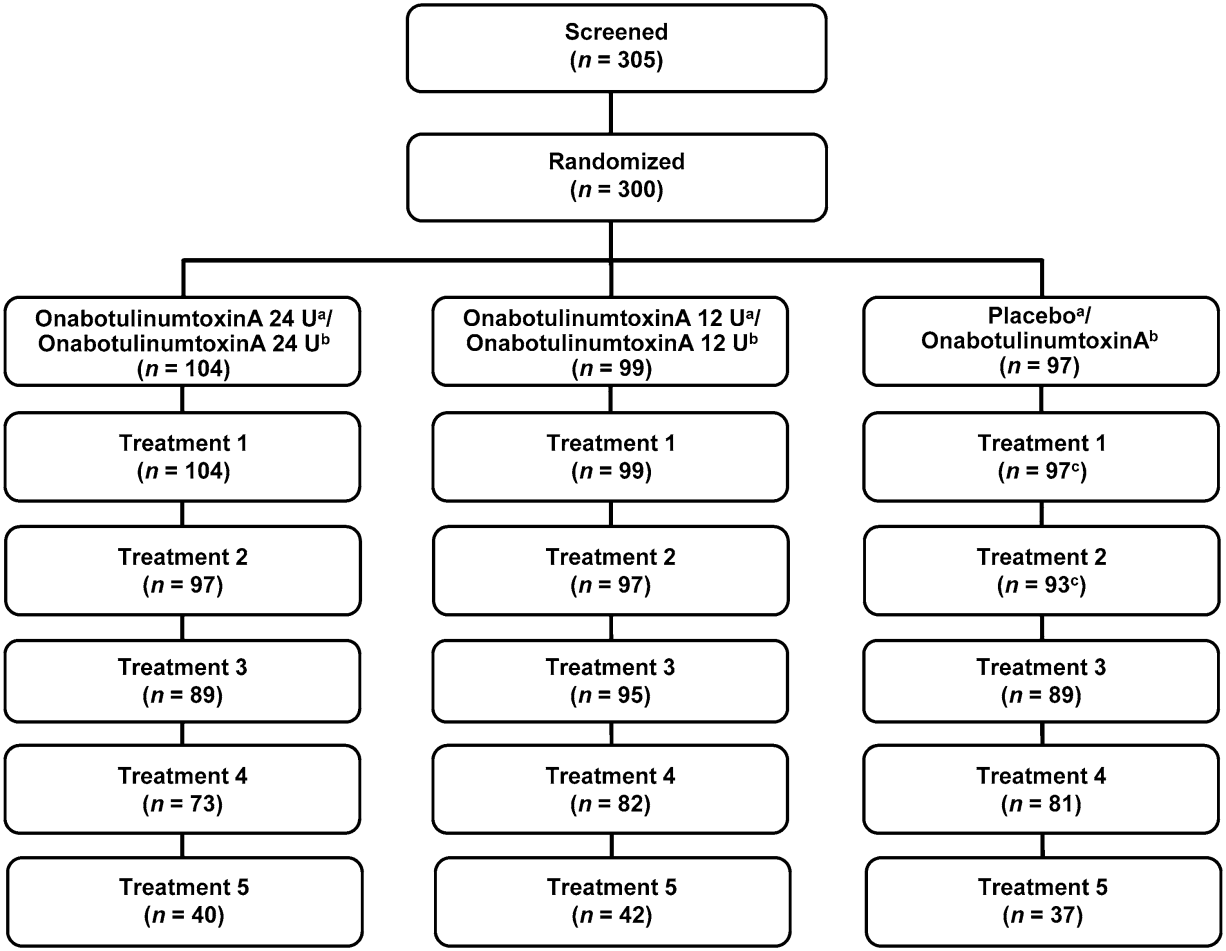
Subject disposition. **a** Randomized treatment assignment in Period 1. **b** Randomized treatment assignment in Period 2. **c** Received placebo

Of the 305 subjects screened, 300 were enrolled and were randomized (Fig. 2). The majority of subjects completed the study (89.3%). Demographic characteristics between treatment groups were similar (Table 2). Mean (range) age was 49.7 (25–64) years; 56.3% of subjects were younger than 50 years of age, and the majority of subjects (74.7%) were female. At baseline, CFL mean severity score at maximum smile was moderate in 49.0% of subjects and severe in 51.0% of subjects. No statistically significant differences in subject-reported measures were noted between groups at baseline.

**Table 2:** Baseline demographics and characteristics

Parameter	OnabotulinumtoxinA 24 U^a^/OnabotulinumtoxinA 24 U^b^ (*n* *=* 104)	OnabotulinumtoxinA 12 U^a^/OnabotulinumtoxinA 12 U^b^ (*n* *=* 99)	Placebo^a^/OnabotulinumtoxinA 24 U^b^ (*n* *=* 48)	Placebo^a^/OnabotulinumtoxinA 12 U^b^ (*n* *=* 49)	Total (*N* *=* 300)
Age (years)					
Mean (SD)	50.2 (6.05)	50.0 (6.11)	49.3 (7.24)	48.3 (8.10)	49.7 (6.64)
≤50, *n* (%)	53 (51.0)	58 (58.6)	28 (58.3)	30 (61.2)	169 (56.3)
Gender, *n* (%)					
Female	84 (80.8)	70 (70.7)	36 (75.0)	34 (69.4)	224 (74.7)
Investigator’s FWS-A assessment of CFL severity at maximum smile					
Moderate	51 (49.0)	49 (49.5)	23 (47.9)	24 (49.0)	147 (49.0)
Severe	53 (51.0)	50 (50.5)	25 (52.1)	25 (51.0)	153 (51.0)
Investigator’s FWS-A assessment of CFL severity at rest					
None	5 (4.8)	5 (5.1)	8 (16.7)	8 (16.3)	26 (8.7)
Mild	61 (58.7)	56 (56.6)	23 (47.9)	24 (49.0)	164 (54.7)
Moderate	32 (30.8)	33 (33.3)	15 (31.3)	12 (24.5)	92 (30.7)
Severe	6 (5.8)	5 (5.1)	2 (4.2)	5 (10.2)	18 (6.0)
Subject assessment of satisfaction with appearance					
Mean (SD)	1.8 (0.55)	2.0 (0.65)	1.8 (0.60)	1.8 (0.63)	1.9 (0.61)
Median	2.0	2.0	2.0	2.0	2.0
Min, max	1, 3	1, 3	1, 3	1, 3	1, 3

*CFL* crow’s feet lines, *FWS-A* Facial Wrinkle Scale with Asian Photonumeric Scale

^a^Randomized treatment assignment in Period 1

^b^Randomized treatment assignment in Period 2

### Efficacy

**Fig. 3:**
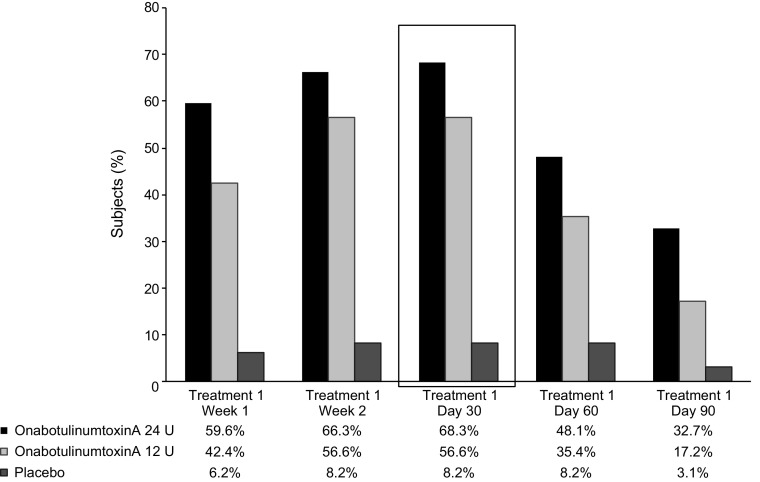
Proportion of subjects achieving none or mild severity of crow’s feet lines at maximum smile, through day 90 of first treatment (intent-to-treat population). *P* < 0.001 at every time point for both onabotulinumtoxinA 24 U & 12 U versus placebo

**Fig. 4:**
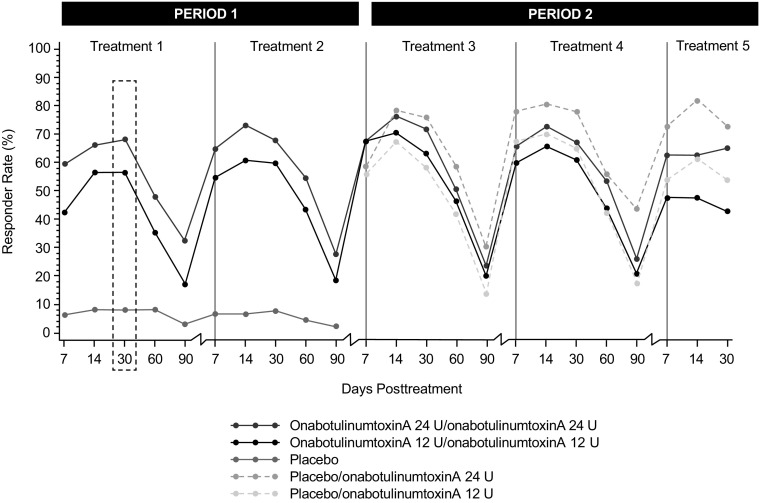
Proportion of subjects achieving none or mild severity of crow’s feet lines at maximum smile, through day 90 of each treatment using the FWS-A (intent-to-treat population). *FWS-A,* Facial Wrinkle Scale with Asian Photonumeric Guide

For the primary endpoint, responder rates at day 30 of treatment period 1 were significantly greater (*P* < 0.001) in the onabotulinumtoxinA 24 and 12 U groups than in the placebo group (Fig. 3). Differences between the onabotulinumtoxinA 24 and 12 U groups were not statistically significant. The proportion of responders was significantly greater (*P* ≤ 0.001) in the onabotulinumtoxinA 24 and 12 U groups compared with the placebo group at all other time points through day 90 in the treatment period 1 (Fig. 4). During the non-placebo-controlled period (treatments 3, 4, and 5), the proportion of responders was similar to that observed during the placebo-controlled treatments. For all treatments (1–5), the proportion of responders in the onabotulinumtoxinA 24 U group was generally greater compared with the onabotulinumtoxinA 12 U group.

**Fig. 5:**
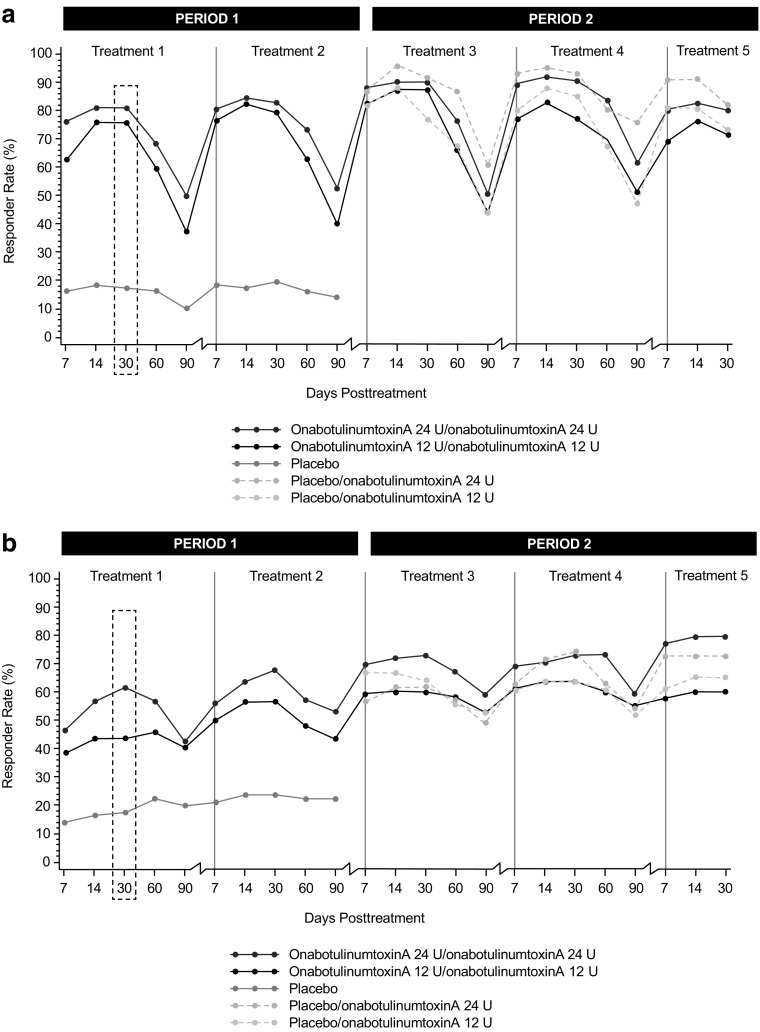
Proportion of subjects achieving at least a 1-grade improvement in severity of crow’s feet lines at maximum smile (a) and at rest (b), through day 90 of each treatment using the FWS-A (intent-to-treat population). *FWS-A,* Facial Wrinkle Scale with Asian Photonumeric Guide

The proportion of responders defined as those achieving at least a 1-grade improvement in severity at maximum smile was significantly greater (*P* < 0.001) in the onabotulinumtoxinA 24 and 12 U groups (80.8 and 75.8%, respectively) compared with the placebo group (17.5%) at day 30. Differences between each onabotulinumtoxinA group and the placebo group were significant at all time points for treatments 1 and 2 (through day 90; *P* ≤ 0.001) (Fig. 5a). Similarly, at day 30 of treatment period 1, among the 274 subjects with at least mild static CFL at baseline, a greater proportion of subjects achieved at least a 1-grade improvement in CFL severity at rest with onabotulinumtoxinA 24 U (61.6%) and onabotulinumtoxinA 12 U (43.6%) compared with placebo (17.3%; *P* < 0.001, both comparisons) (Fig. 5b). Differences were significant (*P* ≤ 0.003) at all time points in treatment period 1 (up to 90 days). For treatment period 2, the proportion of responders in the onabotulinumtoxinA groups was similar to that observed during the placebo-controlled treatment (period 1), both at maximum smile and at rest. Responder rates were numerically higher in the onabotulinumtoxinA 24 U group than in the 12 U group, but differences were not statistically significant at any time point across treatments.

**Fig. 6:**
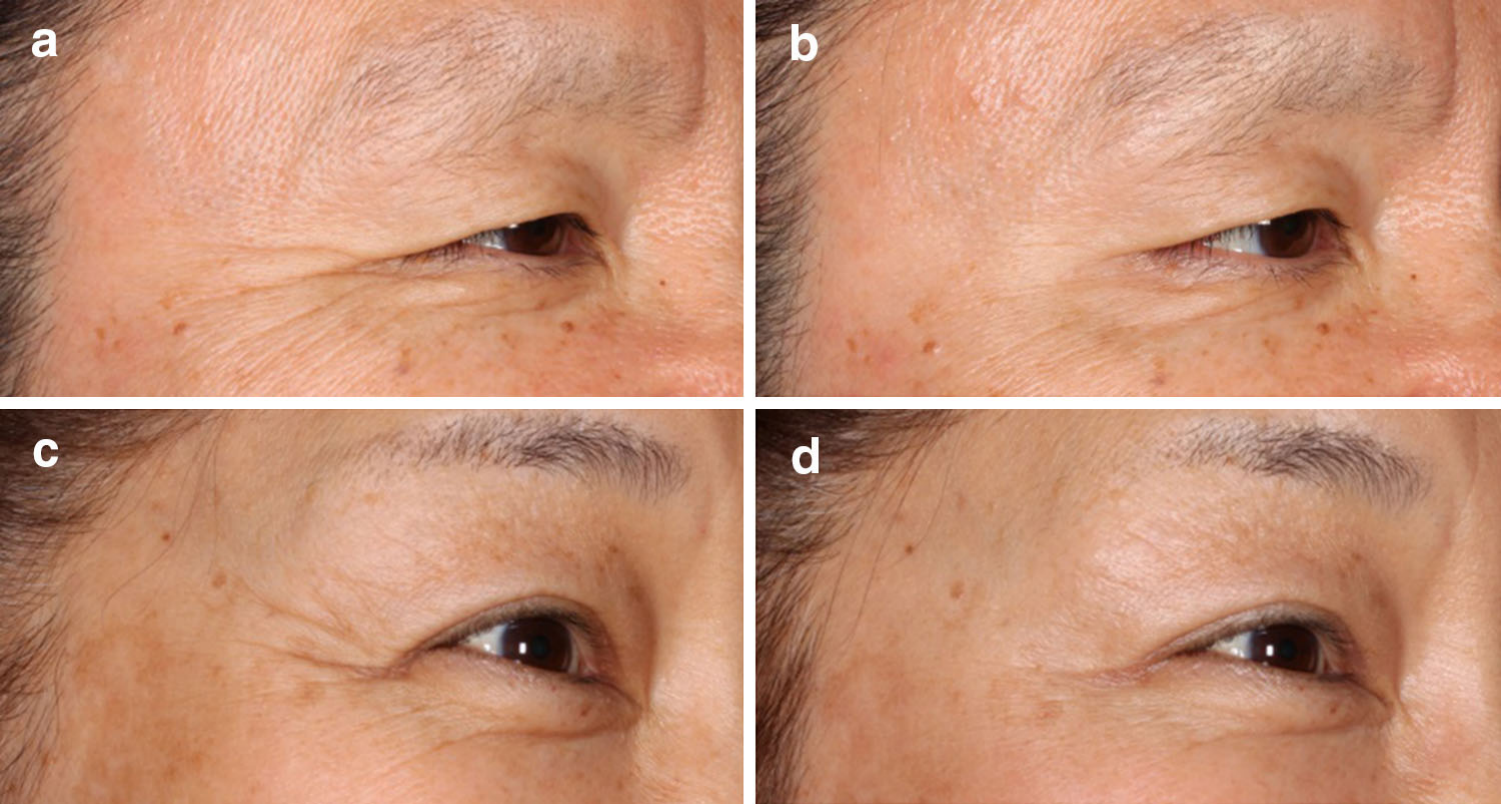
Representative photographs of Subject 1 and Subject 2 taken at baseline (A, C) and at the end of treatment period 1 on day 30 (B, D). Subject 1, a 53-year-old Japanese female, received onabotulinumtoxinA 12 U, while Subject 2, a 55-year-old Japanese female, received onabotulinumtoxinA 24 U

Representative photographs of 2 subjects at baseline and at the end of treatment period 1 are shown in Fig. 6. Both female subjects had severe CFL at baseline that improved to mild at 30 days after treatment with onabotulinumtoxinA 24 U or 12 U.

#### Duration of Effect

The median duration of effect observed for treatment 1 with onabotulinumtoxinA 24 and 12 U, respectively, using the responder definition of none or mild CFL severity at maximum smile at day 30 was approximately 3 months (95.0 and 85.0 days). For the analyses using the responder definition of at least a 1-grade improvement in CFL at maximum smile, the median duration of effect was up to 4 months (118.0 and 92.0 days in the onabotulinumtoxinA 24 and 12 U groups, respectively). The median duration of effect using the responder definition of at least a 1-grade improvement in CFL at rest was at least 4 months (120.0 and 155.0 days in the onabotulinumtoxinA 24 and 12 U groups, respectively).

#### Subject Assessments

Subject-assessed measures at day 30 of treatment 1 are summarized in Table 3. At day 30, the proportion of subjects who reported being very satisfied or satisfied with their CFL appearance on the Subject Assessment of Satisfaction with Appearance was significantly higher for onabotulinumtoxinA 24 and 12 U compared with placebo (*P* < 0.001, both comparisons). Responder rates for onabotulinumtoxinA 24 and 12 U were also significantly greater than placebo at other time points, through day 90 after each treatment in treatment period 1 (*P* ≤ 0.048). In treatment period 2, the proportion of responders in the onabotulinumtoxinA groups was similar to that observed during placebo-controlled treatment period 1.

**Table 3:** Subject-reported outcomes at day 30 of treatment 1

Endpoint	OnabotulinumtoxinA 24 U (*n* *=* 104)	OnabotulinumtoxinA 12 U (*n* *=* 99)	Placebo (*n* *=* 97)
Satisfaction with appearance			
Proportion of responders classified as “very satisfied” or “satisfied” with CFL appearance, *n* (%)	42 (40.4)	28 (28.3)	4 (4.1)
*P* value versus placebo*	<0.001	<0.001	
FLSQ overall satisfaction item			
Proportion of responders classified as “very satisfied” or “mostly satisfied” with CFL appearance, *n* (%)	53 (51.0)	41 (41.4)	7 (7.2)
*P* value versus placebo*	<0.001	<0.001	
SGA-CFL			
Proportion of responders classified as “very much improved” or “much improved,” *n* (%)	51 (49)	39 (39.4)	1 (1.0)
*P* value versus placebo*	<0.001	0.002	
SPA			
Subjects who rated themselves younger than at baseline,^a^ *n* (%)	32 (32.0)	29 (30.5)	7 (7.6)
*P* value versus placebo*	<0.001	<0.001	
FLO-11 responders (psychological impact items)			
Item 2 (“look older”): ≥2-point improvement from baseline,^b^ *n* (%)	59 (57.3)	53 (54.6)	31 (32.6)
*P* value versus placebo*	<0.001	0.002	
Item 5 (“look less attractive”): ≥2-point improvement from baseline,^b^ *n* (%)	52 (51.0)	48 (50.0)	20 (21.1)
P value versus placebo*	<0.001	<0.001	
Item 8 (“look tired”): ≥3-point improvement from baseline,^c^ *n* (%)	40 (40.0)	37 (41.1)	21 (23.6)
*P* value versus placebo*	0.018	0.012	

*CFL* crow’s feet lines, *FLO-11* 11-item Facial Line Outcomes questionnaire, *FLSQ* Facial Line Satisfaction Questionnaire, *FWS-A* Facial Wrinkle Scale with Asian Photonumeric Scale, *SGA-CFL* Subject’s Global Assessment with crow’s feet lines, *SPA* Self-Perception of Age

* *P* values for between-treatment comparisons were determined by Cochran–Mantel–Haenszel tests stratified by baseline CFL severity at maximum smile as assessed using the FWS-A

^a^Only subjects who rated themselves as looking their current age or older at baseline are included in the analysis. Subjects were considered responders if they rated themselves from “look my current age” at baseline to “look younger’ or from “look older” at baseline to “look my current age/younger.”

^b^Only subjects with baseline scores ≥2 are included

^c^Only subjects with baseline scores ≥3 are included

The proportion of responders who reported being very satisfied or mostly satisfied with treatment on the FLSQ overall satisfaction item at day 30 was significantly higher for onabotulinumtoxinA 24 and 12 U than with placebo (*P* < 0.001, both comparisons). Responder rates for onabotulinumtoxinA 24 and 12 U were also significantly greater than placebo at other time points, through day 90 after each treatment in treatment period 1 (*P* ≤ 0.002). In treatment period 2, the proportion of responders among subjects treated with onabotulinumtoxinA was similar to that observed during placebo-controlled treatment period 1.

The proportion of SGA-CFL responders at day 30 who were very much or much improved was significantly higher for onabotulinumtoxinA 24 and 12 U than with placebo (*P* < 0.001, both comparisons). Additionally, the proportion of subjects at day 30 who considered themselves as looking younger was significantly higher for onabotulinumtoxinA 24 and 12 U than with placebo (*P* < 0.001, both comparisons). Finally, FLO-11 items that assess psychological impact showed significantly greater improvement at day 30 with onabotulinumtoxinA 24 and 12 U than with placebo (*P* ≤ 0.018, all comparisons). The proportion of responders among subjects treated in the onabotulinumtoxinA 24 U group was numerically greater compared with those in the 12 U group for all subject-reported outcomes, except item 8 of the FLO-11 questionnaire; more subjects in the onabotulinumtoxinA 24 U group rated themselves as looking younger on the SPA instrument compared with subjects in the 12 U group (Table 3).

Based on the subjects’ perception of onset of improvement in their CFL severity, the median onset of effect in the onabotulinumtoxinA groups was 3 days in treatment period 1. The median onset of effect was similar (3 days or 2 days) in treatment period 2. All other subject-reported measures were achieved with statistical significance favoring onabotulinumtoxinA treatment at day 30 and lasting through at least day 60 of each treatment.

### Safety

Repeated treatment with onabotulinumtoxinA 24 and 12 U in the CFL areas (up to 5 treatments over 13 months) was safe and well tolerated. Over the entire study, a greater proportion of subjects who received onabotulinumtoxinA (47.0% [71/151] for 24 U; 59.4% [85/143] for 12 U) experienced 1 or more treatment-emergent AEs (TEAEs) compared with subjects who received placebo (35.1% [34/97]), likely owing to the greater exposure to onabotulinumtoxinA (up to 5 treatments) compared with placebo (up to 2 treatments). The proportion of subjects experiencing TEAEs was comparable between all treatment groups during the placebo-controlled period 1 (25.2% [38/151] for 24 U; 29.4% [42/143] for 12 U; 22.7% [22/97] for placebo).

The most common TEAEs (incidence ≥1%) across all treatment groups are displayed in Table 4. All TEAEs reported during the study were mild or moderate in severity. Treatment-related AEs occurred in 13 subjects (4.0% [6/151] with onabotulinumtoxinA 24 U, 2.8% [4/143] with onabotulinumtoxinA 12 U, and 3.1% [3/97] with placebo). The most frequently reported treatment-related AEs were malaise (*n* *=* 2, 24 U and placebo), headache (*n* *=* 2, placebo), and sensory disturbance (*n* *=* 2, 12 U and placebo); all other treatment-related AEs occurred in 1 onabotulinumtoxinA subject each (abnormal sensation in eye, eyelid edema, eyelid pain, eyelid ptosis, abdominal pain upper, thirst, injection site pain, injection site warmth, injection site pruritus, dysphoria, brow ptosis, skin tightening); some subjects experienced more than 1 treatment-related AE. All treatment-related AEs were mild in severity.

**Table 4:** Most common TEAEs (Incidence ≥1%) for entire study

System organ class	Preferred term	OnabotulinumtoxinA 24 U (*n* *=* 151) *n* (%)	OnabotulinumtoxinA 12 U (*n* *=* 143) *n* (%)	OnabotulinumtoxinA Total (*n* *=* 294) *n* (%)	Placebo (*n* *=* 97) *n* (%)
Overall		71 (47.0)	85 (59.4)	156 (53.1)	34 (35.1)
Infections and infestations	Nasopharyngitis	23 (15.2)	39 (27.3)	62 (21.1)	5 (5.2)
	Gastroenteritis	3 (2.0)	2 (1.4)	5 (1.7)	0 (0.0)
	Oral herpes	2 (1.3)	4 (2.8)	6 (2.0)	0 (0.0)
	Herpes zoster	2 (1.3)	0 (0.0)	2 (0.7)	0 (0.0)
	Bronchitis	1 (0.7)	2 (1.4)	3 (1.0)	1 (1.0)
	Folliculitis	1 (0.7)	1 (0.7)	2 (0.7)	2 (2.1)
	Cystitis	1 (0.7)	1 (0.7)	2 (0.7)	1 (1.0)
	Cellulitis	1 (0.7)	1 (0.7)	2 (0.7)	0 (0.0)
	Pulpitis dental	0 (0.0)	0 (0.0)	0 (0.0)	1 (1.0)
General disorders and administration site conditions	Injection site hemorrhage	6 (4.0)	7 (4.9)	13 (4.4)	1 (1.0)
	Injection site bruising	6 (4.0)	3 (2.1)	9 (3.1)	1 (1.0)
	Malaise	1 (0.7)	0 (0.0)	1 (0.3)	1 (1.0)
	Puncture site pain	0 (0.0)	0 (0.0)	0 (0.0)	1 (1.0)
	Pyrexia	0 (0.0)	0 (0.0)	0 (0.0)	1 (1.0)
Injury, poisoning and procedural complications	Ligament sprain	5 (3.3)	2 (1.4)	7 (2.4)	0 (0.0)
	Contusion	2 (1.3)	5 (3.5)	7 (2.4)	1 (1.0)
	Excoriation	3 (2.0)	3 (2.1)	6 (2.0)	0 (0.0)
	Procedural pain	1 (0.7)	2 (1.4)	3 (1.0)	0 (0.0)
	Road traffic accident	1 (0.7)	2 (1.4)	3 (1.0)	0 (0.0)
Nervous system disorders	Headache	4 (2.6)	3 (2.1)	7 (2.4)	3 (3.1)
	Hypoesthesia	2 (1.3)	0 (0.0)	2 (0.7)	0 (0.0)
	Sensory disturbance	1 (0.7)	1 (0.7)	2 (0.7)	1 (1.0)
Musculoskeletal and connective tissue disorders	Back pain	3 (2.0)	2 (1.4)	5 (1.7)	1 (1.0)
	Musculoskeletal stiffness	2 (1.3)	1 (0.7)	3 (1.0)	0 (0.0)
	Tenosynovitis	1 (0.7)	2 (1.4)	3 (1.0)	1 (1.0)
	Musculoskeletal pain	0 (0.0)	1 (0.7)	1 (0.3)	1 (1.0)
	Plantar fasciitis	0 (0.0)	1 (0.7)	1 (0.3)	1 (1.0)
Skin and subcutaneous tissue disorders	Purpura	2 (1.3)	6 (4.2)	8 (2.7)	1 (1.0)
	Xeroderma	2 (1.3)	2 (1.4)	4 (1.4)	0 (0.0)
	Eczema	2 (1.3)	1 (0.7)	3 (1.0)	1 (1.0)
	Acne	1 (0.7)	1 (0.7)	2 (0.7)	2 (2.1)
	Urticaria	0 (0.0)	2 (1.4)	2 (0.7)	1 (1.0)
	Dry skin	0 (0.0)	2 (1.4)	2 (0.7)	0 (0.0)
	Hemorrhage subcutaneous	0 (0.0)	2 (1.4)	2 (0.7)	0 (0.0)
	Idiopathic urticaria	0 (0.0)	2 (1.4)	2 (0.7)	0 (0.0)
	Skin erosion	0 (0.0)	0 (0.0)	0 (0.0)	1 (1.0)
	Skin hypopigmentation	0 (0.0)	0 (0.0)	0 (0.0)	1 (1.0)
Eye disorders	Dry eye	2 (1.3)	2 (1.4)	4 (1.4)	1 (1.0)
	Conjunctivitis allergic	2 (1.3)	1 (0.7)	3 (1.0)	1 (1.0)
	Eyelid sensory disorder	2 (1.3)	1 (0.7)	3 (1.0)	0 (0.0)
	Abnormal sensation in eye	0 (0.0)	2 (1.4)	2 (0.7)	0 (0.0)
	Vision blurred	0 (0.0)	1 (0.7)	1 (0.3)	1 (1.0)
Immune system disorders	Seasonal allergy	2 (1.3)	2 (1.4)	4 (1.4)	0 (0.0)
Respiratory, thoracic and mediastinal disorders	Rhinitis allergic	2 (1.3)	1 (0.7)	3 (1.0)	0 (0.0)
	Upper respiratory tract inflammation	2 (1.3)	1 (0.7)	3 (1.0)	0 (0.0)
	Oropharyngeal pain	1 (0.7)	0 (0.0)	1 (0.3)	3 (3.1)
	Asthma	0 (0.0)	2 (1.4)	2 (0.7)	0 (0.0)
	Pneumothorax spontaneous	0 (0.0)	0 (0.0)	0 (0.0)	1 (1.0)
Gastrointestinal disorders	Gingival swelling	2 (1.3)	0 (0.0)	2 (0.7)	0 (0.0)
	Dental caries	1 (0.7)	3 (2.1)	4 (1.4)	1 (1.0)
	Gastritis	0 (0.0)	3 (2.1)	3 (1.0)	1 (1.0)
	Diarrhea	0 (0.0)	2 (1.4)	2 (0.7)	1 (1.0)
	Gastroesophageal reflux disease	0 (0.0)	2 (1.4)	2 (0.7)	1 (1.0)
	Inguinal hernia	0 (0.0)	1 (0.7)	1 (0.3)	1 (1.0)
	Dyspepsia	0 (0.0)	0 (0.0)	0 (0.0)	1 (1.0)
	Gastric polyps	0 (0.0)	0 (0.0)	0 (0.0)	1 (1.0)
	Nausea	0 (0.0)	0 (0.0)	0 (0.0)	1 (1.0)
Neoplasms benign, malignant and unspecified (includes cysts and polyps)	Skin papilloma	1 (0.7)	0 (0.0)	1 (0.3)	1 (1.0)
	Cervicitis human papilloma virus	0 (0.0)	1 (0.7)	1 (0.3)	0 (0.0)
Investigations	Weight decreased	0 (0.0)	0 (0.0)	0 (0.0)	1 (1.0)
Ear and labyrinth disorders	Vertigo	0 (0.0)	2 (1.4)	2 (0.7)	0 (0.0)

Four subjects (1 with onabotulinumtoxinA 24 U; 2 with onabotulinumtoxinA 12 U; 1 with placebo) experienced events that were identified as possible spread of toxin: blurred vision (1 subject each with onabotulinumtoxinA 12 U and placebo); eyelid ptosis (1 subject with onabotulinumtoxinA 24 U); and muscular weakness (1 subject with onabotulinumtoxinA 12 U). Safety adjudication of each event determined that the events of blurred vision and eyelid ptosis in onabotulinumtoxinA subjects were local events consistent with the known pharmacology of onabotulinumtoxinA, while the events of blurred vision (with placebo) and muscle weakness (with onabotulinumtoxinA 12 U) did not represent the distant spread of toxin.

Two subjects were discontinued from the study after receiving onabotulinumtoxinA 24 U because of TEAEs considered unrelated to study drug (back pain and angina pectoris). No subject died during the study. Seven subjects experienced a serious TEAE (2.0% [3/151] with onabotulinumtoxinA 24 U, 2.1% [3/143] with onabotulinumtoxinA 12 U, and 1.0% [1/97] with placebo). None of the serious TEAEs were considered to be related to the study drug by the investigators. No clinically meaningful findings were reported from the neurologic assessment. All subjects were negative for neutralizing antibodies at baseline and at the end of the study.

## Discussion

In this pivotal phase 3 study of onabotulinumtoxinA in Japanese subjects, treatment with onabotulinumtoxinA 24 U and 12 U for up to 5 treatments over 13 months was effective in reducing the severity of moderate to severe CFL. Improvements were observed as early as week 1 of treatment 1 and throughout both treatment periods. Overall, the results indicate that a greater proportion of onabotulinumtoxinA-treated subjects experienced improvement in the appearance of their CFL compared with placebo subjects, by all responder definitions, as assessed by the investigator using the FWS-A. The magnitude and time course of effects of onabotulinumtoxinA on responder rates for improvement in CFL severity, as assessed at maximum smile, was consistent over both treatment periods. Indeed, the proportion of responders in treatment cycle 3 was similar among treatment-naive subjects and those who received onabotulinumtoxinA in treatment period 1.

Subject-assessed satisfaction and perception of improvement were consistent with the assessments of the investigators. Significant differences (*P* < 0.001, onabotulinumtoxinA 24 and 12 U vs placebo) in the duration of effect and in the SGA-CFL and SPA favored onabotulinumtoxinA, as reported by subjects; this underscores the clinically meaningful benefit experienced by onabotulinumtoxinA-treated subjects. These results were complemented by significant improvements in the level of satisfaction with appearance and satisfaction with study drug treatment in onabotulinumtoxinA-treated subjects versus placebo subjects. In addition, the median duration of effect with onabotulinumtoxinA treatment was consistent with that reported in previous studies of CFL treatment with onabotulinumtoxinA [7].

Repeated treatment with onabotulinumtoxinA 24 and 12 U, with up to 5 treatment cycles over 13 months, was safe and well tolerated, with no new safety findings or cumulative deleterious effects. Few TEAEs considered related to treatment, serious TEAEs, or discontinuations because of TEAEs were reported. This safety profile was consistent and reproducible across treatments.

The proportion of subjects achieving CFL severity of none or mild at maximum smile with onabotulinumtoxinA 24 U at day 30 (68.3%) is consistent with that observed in a phase 3 study conducted in non-Asians (66.7%) [2]. Duration of effect based on the responder definition of none or mild in CFL severity at maximum smile was 95 days in this study of Japanese subjects and 118 days in the previous non-Asian study. Duration of effect using the definition of at least a 1-grade improvement at maximum smile (118 days) was consistent with findings from the non-Asian study (125 days) [2]. The efficacy of onabotulinumtoxinA in Japanese subjects has been demonstrated for the treatment of glabellar lines [8]. Our results, along with the established efficacy for treatment of glabellar lines, reinforce that onabotulinumtoxinA is a safe and effective esthetic treatment in Japanese subjects [8].

Certain study limitations deserve mention. Because of the frequency of re-treatment, the number of subjects in each treatment group decreased considerably after day 90, potentially limiting data interpretation beyond day 90. Additionally, only placebo-controlled treatment 1 in treatment period 1 was analyzed to assess the duration of effect. Because the study design allowed re-treatment to occur without a required return to baseline and as early as day 90, the duration of effect may have been shortened.

In conclusion, results indicate that repeated treatment with onabotulinumtoxinA (24 and 12 U) is effective and well tolerated in improving the appearance of CFL by reducing CFL severity at maximum smile in Japanese adults, with no new safety findings identified.
